# PERSON-CENTERED CARE: WHY TAKING INDIVIDUALS' CARE PREFERENCES INTO ACCOUNT MATTERS

**DOI:** 10.1093/geroni/igac059.1861

**Published:** 2022-12-20

**Authors:** Frances Hawes, Jane Tavares, Marc Cohen, Ann Hwang

**Affiliations:** University of Wisconsin-Eau Claire, Eau Claire, Wisconsin, United States; University of Massachusetts Boston, Boston, Massachusetts, United States; University of Massachusetts Boston, Boston, Massachusetts, United States; Harvard, Cambridge, Massachusetts, United States

## Abstract

Person-centered care has been recognized as an integral part of a high-quality health care system. Utilizing the 2014 to 2018 waves of the Health and Retirement Study, we explore trends in person-centered care among those 50 and older by examining the extent to which they feel their care preferences are being taken into account, that is, that they are being heard by providers. We analyze the impact of not receiving person-centered care on health care utilization, health outcomes, and preventative care utilization. One-third of respondents reported that their care preferences were “sometimes” or “never” considered. Findings show that wealth and racial disparities in person-centered care are worsening over time. From 2014 to 2018, the percentage of non-Hispanic White respondents who reported that their care preferences were never taken into account decreased while the percentage for Hispanic and non-Hispanic Black individuals increased. Similar trends were seen for low-income individuals. Having a usual source of care was associated with a greater likelihood of having care preferences considered as well as significantly better control of chronic condition and greater use of preventive care. When care preferences are not being taken into account, there is less utilization of health care services, less preventive care usage, poorer control of chronic conditions, and increased risk for higher health care costs. These findings highlight the importance of assuring that people feel listened to by health care providers and emphasize a need for strategies to advance person-centered care for people of color and low-income populations.

